# Outcomes of Patients With Active Cancer and COVID-19 in the Intensive-Care Unit: A Multicenter Ambispective Study

**DOI:** 10.3389/fonc.2022.858276

**Published:** 2022-03-10

**Authors:** Henri Plais, Marie Labruyère, Thibault Creutin, Paula Nay, Gaëtan Plantefeve, Romain Tapponnier, Maud Jonas, Nadege Tchikangoua Ngapmen, Loïc Le Guennec, Charles De Roquetaillade, Laurent Argaud, Matthieu Jamme, Cyril Goulenok, Karim Merouani, Maxime Leclerc, Bertrand Sauneuf, Sami Shidasp, Annabelle Stoclin, Aurélie Bardet, Olivier Mir, Nusaibah Ibrahimi, Jean-François Llitjos

**Affiliations:** ^1^ Intensive Care Unit, Gustave Roussy, Université Paris-Saclay, Villejuif, France; ^2^ Department of Intensive Care, Dijon Bourgogne University Hospital, Dijon, France; ^3^ Service de Médecine Intensive and Réanimation, APHP-CUP, Hôpital Cochin, Paris, France; ^4^ Medical Intensive Care Unit, Ambroise Paré Hospital, AP-HP, Boulogne-Billancourt, France; ^5^ Service de Réanimation Polyvalente, Centre Hospitalier Victor Dupouy, Argenteuil, France; ^6^ Medical Intensive Care Unit, Hôpital Jean Minjoz Hospital, Besançon, France; ^7^ Centre Hospitalier Général de Saint-Nazaire, Service de Médecine Intensive Réanimation, Saint-Nazaire, France; ^8^ Intensive Care Unit, Centre Hospitalier de Château-Thierry, Château-Thierry, France; ^9^ Médecine Intensive Réanimation Neurologique, Département de Neurologie, Groupe Hospitalier Pitié-Salpêtrière, Assistance Publique-Hôpitaux de Paris, Paris, France; ^10^ Department of Anesthesiology and Critical Care, Hôpital Lariboisière, FHU PROMICE, DMU Parabol, APHP, Paris, France; ^11^ Medical ICU, Edouard Herriot University Hospital, Lyon, France; ^12^ Intensive Care Unit, Poissy-Saint-Germain-en-Laye Hospital, Poissy, France; ^13^ Medical-Surgical Intensive Care Unit, Ramsay Générale de Santé, Hôpital Privé Jacques Cartier, Massy, France; ^14^ Medical and Surgical Intensive Care Unit, Alençon Hospital, Alençon, France; ^15^ Intensive Care Unit, Centre Hospitalier Mémorial France Etats-Unis, Saint-Lô, France; ^16^ Réanimation - Médecine Intensive, Centre Hospitalier Public du Cotentin, Cherbourg-en-Cotentin, France; ^17^ Intensive Care Unit, Etampes Hospital, Etampes, France; ^18^ Bureau of Biostatistics and Epidemiology, Gustave Roussy, University Paris-Saclay, Villejuif, France and U1018 INSERM Oncostat, University Paris-Saclay, Labeled Ligue Contre le Cancer, Villejuif, France; ^19^ Gustave-Roussy, Département d’oncologie Médicale, Villejuif, France

**Keywords:** COVID-19, cancer, intensive care unit, hematological malignancies, solid tumors

## Abstract

**Background:**

Several studies report an increased susceptibility to SARS-CoV-2 infection in cancer patients. However, data in the intensive care unit (ICU) are scarce.

**Research Question:**

We aimed to investigate the association between active cancer and mortality among patients requiring organ support in the ICU.

**Study Design and Methods:**

In this ambispective study encompassing 17 hospitals in France, we included all adult active cancer patients with SARS-CoV-2 infection requiring organ support and admitted in ICU. For each cancer patient, we included 3 non cancer patients as controls. Patients were matched at the same ratio using the inverse probability weighting approach based on a propensity score assessing the probability of cancer at admission. Mortality at day 60 after ICU admission was compared between cancer patients and non-cancer patients using primary logistic regression analysis and secondary multivariable analyses.

**Results:**

Between March 12, 2020 and March 8, 2021, 2608 patients were admitted with SARS-CoV-2 infection in our study, accounting for 2.8% of the total population of patients with SARS-CoV-2 admitted in all French ICUs within the same period. Among them, 105 (n=4%) presented with cancer (51 patients had hematological malignancy and 54 patients had solid tumors). 409 of 420 patients were included in the propensity score matching process, of whom 307 patients in the non-cancer group and 102 patients in the cancer group. 145 patients (35%) died in the ICU at day 60, 59 (56%) with cancer and 86 (27%) without cancer. In the primary logistic regression analysis, the odds ratio for death associated to cancer was 2.3 (95%CI 1.24 – 4.28, p=0.0082) higher for cancer patients than for a non-cancer patient at ICU admission. Exploratory multivariable analyses showed that solid tumor (OR: 2.344 (0.87-6.31), p=0.062) and hematological malignancies (OR: 4.144 (1.24-13.83), p=0.062) were independently associated with mortality.

**Interpretation:**

Patients with cancer and requiring ICU admission for SARS-CoV-2 infection had an increased mortality, hematological malignancy harboring the higher risk in comparison to solid tumors.

**Graphical Abstract:**
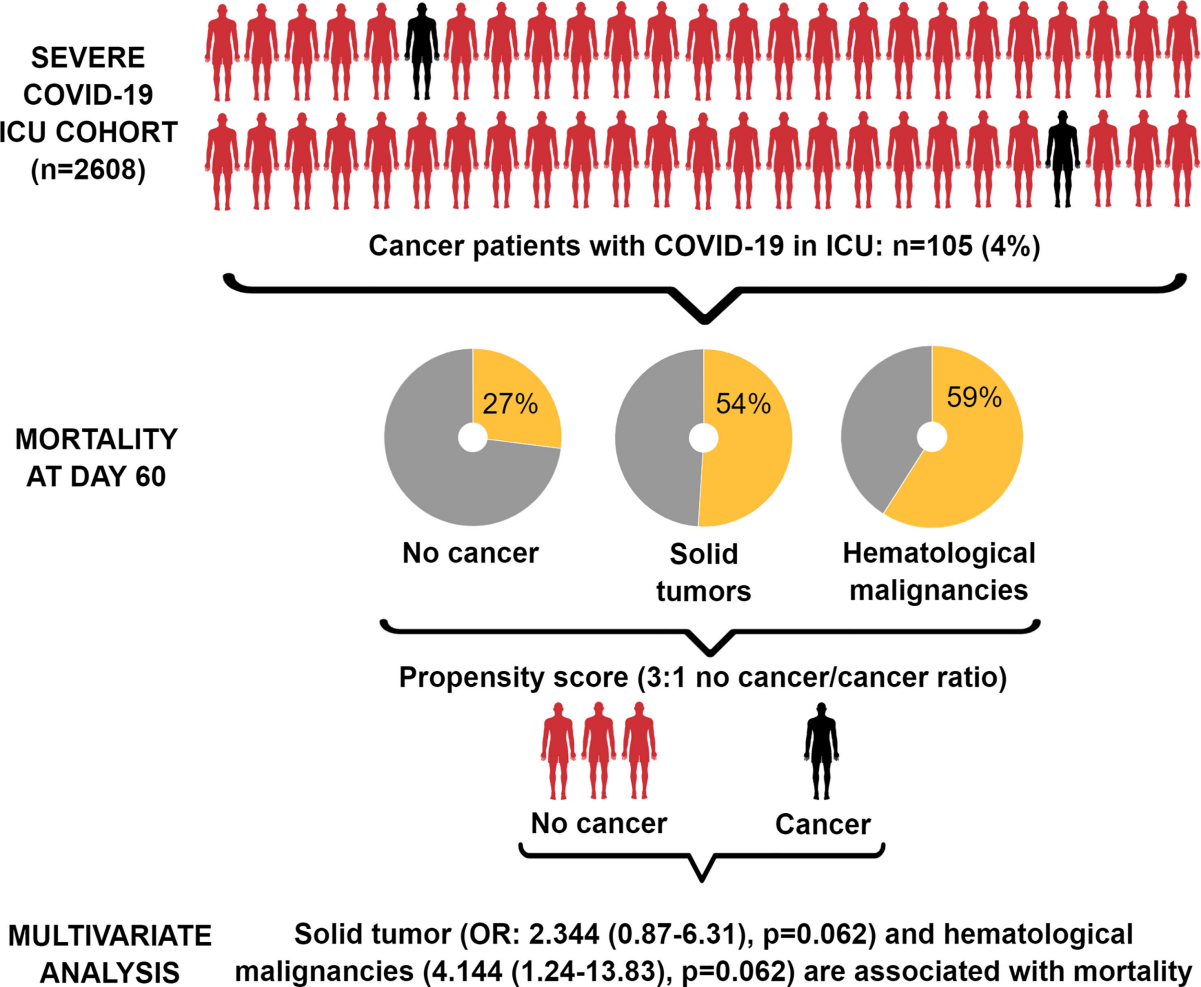


## Background

Ever since it was first reported in China in late 2019, coronavirus disease 2019 (COVID-19) has impacted millions of patients worldwide. It is now well documented that this disease, caused by severe acute respiratory syndrome coronavirus 2 (SARS-CoV-2) infection, preferentially affects the elderly and those with comorbidities (1, 2). Among frail patients, those with cancer are reported to harbor an increased susceptibility to SARS-COV-2 infection (3). This vulnerability is thought to be related to cancer-related immune compromised status and cancer-directed treatments.

However, data regarding the impact of SARS-CoV-2 infection on mortality of cancer patients are conflicting (4, 5). Although data in the ICU setting are scarce, most studies have identified a higher risk of mortality in cancer patients (6). Several confounding factors, such as specific patient case-mix, cancer treatment, or progression status, may explain this discrepancy. Furthermore, considering cancer-related immunosuppression, data regarding ICU-acquired infections that could prolong ICU morbidity and mortality are scarce.

Therefore, this study aimed to determine the outcomes of COVID-19 patients with cancer in the ICU, compared to a cohort of patients without cancer, using the inverse of the propensity score (PS) as weight. This enabled the creation of homogeneous cohorts from a real-life hospital setting with limited confounding effects. Using a large multicenter cohort of severe patients, we aim to provide robust and clinically relevant data to assist clinicians in the emergency management of fragile patients.

## Methods

### Study Design

In this multicentre ambispective cohort study, we enrolled all consecutive adult patients with SARS-COV-2 infection who were admitted to any of the 17 participating French ICUs. The study was approved by the Institutional Review Board (Comité de protection des personnes Ile-de-Frande IV, IRB number 00003835), which waived the requirement for informed consent. Data were collected retrospectively between 12 March 2020 and 4 January 2021 and prospectively between 4 January 2021 and 4 March 2021.

### Study Participants

We included all consecutive adult patients (age >18 years) with laboratory-confirmed SARS-CoV-2 infection who were admitted to a participating ICU. The patients were followed-up until discharge from ICU or death. Active cancer was defined as cancer for which treatment (including chemotherapy, targeted therapy, immunotherapy, radiotherapy, or surgery) had been administered within six months, or with active surveillance within 6 months of ICU admission or haematological cancer that is not in complete remission. In patients with cancer, cancer-specific variables included diagnosis date, type of cancer, oncological treatments, response to treatments, date of last cancer treatment, TNM classification with existence and localisation of metastasis in solid tumors and haematopoietic stem cell transplant (HSCT) in haematological malignancies.

### Definitions

Laboratory confirmation of SARS-COV-2 infection was based on SARS-CoV-2 detection by real-time reverse transcriptase polymerase chain reaction from nasal swabs or lower respiratory tract secretions. Obesity was defined as a body mass index (BMI) > 30 kg/m^2^. In addition to cancer, patients were considered immunocompromised if one or more of the following conditions were observed: solid organ transplantation, human immunodeficiency virus (HIV) infection with or without acquired immunodeficiency syndrome, treatment with corticosteroids (> 3 months at any dosage or ≥ 1 mg/kg prednisone equivalent per day for > 7 days), or treatment with other immunosuppressive drugs. Severity at admission was assessed using the Simplified Acute Physiology Score 2 (SAPS II) and Sequential Organ Failure Assessment (SOFA) scores. Acute respiratory distress syndrome (ARDS) was diagnosed according to the Berlin definition. ICU-acquired pneumonia was defined as a new onset of probable or definite infection not present at the time of ICU admission and that developed after the first 48 h after ICU admission. Only the first episode of ICU-acquired pneumonia was considered in this study.

### Data Collection and Analysis

Data collection was performed in a standardised and de-identified manner using REDCap (Research Electronic Data Capture, electronic data capture tools hosted at Gustave Roussy Cancer Centre). Data collection was performed by a single investigator in each centre, with each institution being assigned a unique number. Tumour types were classified according to the International Classification of Diseases, 10th Revision (ICD-10) codes. Data were divided into the following main categories: demographics and comorbidities, oncological history, clinical and biological data at ICU admission, ICU course, and clinical outcomes (including death and ICU-acquired infections).

### Outcomes

The primary outcome of interest was the association between cancer (either haematological malignancies or solid tumours) and the likelihood of mortality at day 60 after ICU admission. All-cause inpatient fatalities included death as a direct result of SARS-COV-2 infection and any other cause.

### Sampling Process and Sample Size Justification

The cohort of patients with cancer included all patients with either solid tumours or haematological malignancy who were admitted to the ICU of the participating centres between 12 March 2020 and 8 March 2021 with severe SARS-CoV-2 infection. For each patient included in the cancer cohort, each centre included three subsequent patients without cancer. A non-participation register was set to ensure internal (regarding sampling procedure) and external (regarding extrapolations to wider populations) validity. The sample size was calculated based on the principal criterion. The assumption was made to have a mortality rate of 30% in the cohort of non-cancer patients, with a 1:3 design. The 5% bilateral test was based on an odds ratio (OR) of approximately 2, with a power of 80%. No withdrawal of consent or loss of sight was expected in the primary endpoint evaluation. A total of 398 patients were estimated, rounded off to 400 patients (300 in the non-cancer cohort and 100 in the cancer cohort).

### Statistical Analysis

The primary outcome measure was mortality at day 60 after ICU admission. In this observational study, potential confounding bias among included patients was managed using the inverse probability weighting (IPW) method. A PS for cancer (with respect to non-cancer) was built using a wide list of baseline covariates, including sex, age, BMI, diabetes mellitus, chronic respiratory disease, congestive heart failure, organ transplant, immunosuppressive therapy, HIV, antimicrobial treatment, hospitalisation within the last 3 months, SAPS II and SOFA scores, and PaO_2_ at baseline. Subsequently, each patient’s weight was computed using the inverse of the PS for the actual cancer status. Finally, the association between cancer and mortality at day 60 after ICU admission was modelled using logistic regression on the weighted population. ORs, associated robust confidence intervals (CIs), and p-values were provided.

To provide further information on the prognostic factors of death in the ICU, multivariable logistic regressions were estimated, allowing estimation of effect size for additional confounding parameters. To assess the robustness of the main model and the ability of the IPW methodology to manage confounding bias, the characteristics of the weighted population were analysed, and alternative models for PS building or PS inclusion in the final model were tested. Data were analysed using SAS version 9·4 (SAS Institute Inc, Cary, NC, USA).

## Results

Between 12 March 2020 and 8 March 2021, 2,608 patients were admitted with SARS-CoV-2 infection in our study, accounting for 2.8% of the total population of patients with SARS-CoV-2 admitted in all French ICUs during the same period. The main patient characteristics are presented in **Table 1**. The prevalence of cancer at ICU admission was 4% (n=105), with 51 and 54 patients presenting with haematological malignancy and solid tumours, respectively (**Table 2**). Given that 105 patients had cancer at admission among the 2608 patients admitted during the study period, and because we used a 1:3 ratio matching to build the propensity score, we obtained a final cohort of 420 patients which constitutes the core of this work for the mortality analyses (**Supplementary Figure 1**).

**Table 1:** Patients characteristics at admission.

	No cancer (n=315)	Cancer patients (n=105)	Hematological maligancies (n=51)	Solid tumors (n=54)
Sex				
Female	96 (30·6%)	33 (31·4%)	19 (37·3%)	14 (25·9%)
Male	218 (69·4%)	72 (68·6%)	32 (62·7%)	40 (74·1%)
Age, Years	67 (59 - 75)	69 (65 - 73)	69 (64 - 72)	69 (65 - 73)
BMI, kg/m²	28 (25 - 32)	26 (23 - 29)	25 (23 - 28)	26 (23 - 29)
Diabetes mellitus	107 (34·0%)	24 (22·9%)	9 (17·6%)	15 (27·8%)
Chronic kidney disease	32 (10·2%)	12 (11·4%)	8 (15·7%)	4 (7·4%)
Congestive heart failure	29 (9·2%)	10 (9·5%)	4 (7·8%)	6 (11·1%)
Chronic respiratory disease	12 (3·8%)	8 (7·6%)	4 (7·8%)	4 (7·4%)
Cirrhosis (Child B or C)	8 (2·5%)	1 (1·0%)	0 (0%)	1 (1·9%)
Solid organ transplant	11 (3·5%)	1 (1·0%)	0 (0%)	1 (1·9%)
Immunosuppressive agents	17 (5·4%)	26 (24·8%)	18 (35·3%)	8 (14·8%)
HIV	6 (1·9%)	3 (2·9%)	1 (2·0%)	2 (3·7%)
<3 months hospitalization	32 (10·2%)	42 (40·0%)	22 (43·1%)	20 (37·0%)
Recent antimicrobial treatments	83 (26·4%)	50 (47·6%)	27 (52·9%)	23 (42·6%)
Pre-admission location				
Home	127 (40·3%)	31 (29·5%)	13 (25·5%)	18 (33·3%)
Non-ICU units	157 (49·8%)	59 (56·2%)	30 (58·8%)	29 (53·7%)
Intensive car units	31 (9·8%)	15 (14·3%)	8 (15·7%)	7 (13·0%)
SAPS II score	36 (28 - 45)	43 (36 - 58)	47 (37 - 58)	41 (35 - 56)
SOFA score	4 (3 - 7)	6 (3 - 8)	6 (4 - 8)	4 (3 - 8)
SARS-Cov-2 diagnosis				
PCR	306 (97·8%)	100 (96·2%)	50 (98·0%)	50 (94·3%)
Serology	2 (0·6%)	1 (1·0%)	0 (0%)	1 (1·9%)
CT-scan	5 (1·6%)	3 (2·9%)	1 (2·0%)	2 (3·8%)
PaO_2_ at admission	70 (59 - 85)	67 (58 - 82)	67 (56 - 93)	66 (59 - 77)
pH at admission	7·45 (7·40 - 7·48)	7·45 (7·41 - 7·48)	7·45 (7·40 - 7·48)	7·45 (7·42 - 7·48)
Lactate at admission	1·3 (1·0 - 1·8)	1·4 (1·0 - 2·0)	1·2 (1·0 - 1·9)	1·5 (1·1 - 2·1)

BMI, Body mass index; HIV, Human immunodeficiency virus; ICU, intensive care units; SAPS II score, simplified acute physiology score; SOFA score, Sepsis-related Organ Failure Assessment; PCR, polymerase chain reaction; CT, computed tomography.

**Table 2:** Characteristics of hematological malignancies and solid cancer.

Hematological malignancies	Total (n=51)
Type of hematological malignancies	
Lymphoma/Chronic lymphocytic leukemia	27 (52·9%)
Myeloma	18 (35·3%)
Acute myeloid leukemia	3 (5·9%)
Myeloproliferative disorders	3 (5·9%)
Status prior to admission	
Diagnosis without treatment	9 (18·0%)
Ongoing treatment	20 (40·0%)
Treated with favourable response	13 (26·0%)
Treated without favourable response	8 (16·0%)
Haematopoietic stem cell transplantation (HSCT)	
None	41 (80·4%)
Allogeneic HSCT	1 (2·0%)
Autologous HSCT	9 (17·6%)
Hematological maligancy treatment in the last 4 weeks	
No	24 (47·1%)
Yes	27 (52·9%)
Solid tumors	Total (n=54)
Solid tumor type	
Breast	6 (11·1%)
Colorectal	10 (18·5%)
Lung	12 (22·2%)
Prostate	9 (16·7%)
Digestive organs, non colorectal	8 (14·8%)
Urinary tract	2 (3·7%)
Female genital organs	2 (3·7%)
Central nervous system	1 (1·9%)
Skin	2 (3·7%)
Endocrine system	2 (3·7%)
Metastasis	23 (43·4%)
Metastasis location	
Central nervous system	6 (13·0%)
Ganglionnary	12 (26·1%)
Lung	18 (39·1%)
Pleural	6 (13·0%)
Abdomen, non liver	6 (13·0%)
Liver	16 (34·8%)
Endocrine system	6 (13·0%)
Others	10 (21·7%)
Status prior to admission	
Diagnosis without treatment	12 (22·2%)
Ongoing treatment	25 (46·3%)
Treated with favourable response	14 (25·9%)
Treated without favourable response	3 (5·6%)
Solid tumor treatment in the last 4 weeks	
No	27 (50·0%)
Yes	27 (50·0%)

In our study, solid organ transplant was significantly associated with less cancer (OR: 0·01, 95% CI: 0–0·17, p<0·0001), whereas immunosuppressive agents (OR: 11·56, 95% CI: 4·2–31·81, p<0·0001) and hospitalisation in the prior 3 months (OR: 4·03, 95% CI: 1·97–8·26, p>0·0001) were both significantly associated with a higher risk of cancer. From the 420 evaluable severe COVID-19 patients, 409 patients were included in the main analysis, of whom 307 patients were in the non-cancer group and 102 patients in the cancer group. After IPW, the characteristics of the populations were well balanced (**Supplementary Figure 2**).

The mortality rate of patients without cancer was 27%, while that of patients with cancer was 56% (n=59/105). Upon performing logistic regression for death in the weighted cohort, including both patients with and without cancer, we found that cancer was significantly associated with death, with an OR estimate of 2·30 (95% CI: 1·24–4·27, p=0·0082). Accordingly, other factors being equal, the death risk was 2·3 fold greater for cancer patients than for those without cancer at ICU admission.

The extent of this association may be related to the type of cancer, i.e., haematological malignancies or solid tumours. Therefore, we performed a logistic regression analysis of death in the weighted cohort considering the type of cancer. We found that haematological malignancy was significantly associated with death (OR: 3·19, 95% CI: 1·38–7·37, p=0·0065), whereas solid tumours were not associated with death (OR: 1·88, 95% CI: 0·82–4·27, p=0·13). We obtained similar results when patients were balanced using a specific PS (data not shown).

We also assessed the robustness of the estimates by performing sensitivity analyses. Cancer was still associated with ICU mortality when computing the PS using a backward selection with only the significant variables (OR: 2·24, 95% CI: 1·16–4·33, p=0·016) or when the PS was added in the final logistic regression analysis (OR: 2·42, 95% CI: 1·22–10·33, p=0·0019). No parameter was significantly associated with ICU mortality when variables of the PS were included in the final logistic regression model of the weighted population. Including PS components in the final model, we observed a strong trend towards an association between age at admission and ICU mortality (OR: 1·04, 95% CI: 0·996–1·088, p=0·07).

We performed exploratory analyses to determine the association between death and patient characteristics, and ICU management. Although the management of SARS-CoV-2 infection varied among patients (**Table 3**), some parameters were associated with ICU mortality (**Table 4**). In patients who required the use of norepinephrine during the ICU stay (OR: 2·61, 95% CI: 1·10-6·16, p=0·028) and in those who required the use of antiobiotic therapy (OR: 6·85, 95% CI: 2·13-22·02; p=0·0013), mortality was significantly higher. Conversely, treatment with hydroxychloroquine and azithromycin was associated with a better outcome (OR: 0·187, 95% CI: 0·047–0·74; p=0·0177). Prone positioning had a trend towards an association with an unfavourable outcome (OR: 1·99, 95% CI: 0·89–4·44, p=0·092). Of note, we have analysed ICU mortality between pre-treated cancer, non pre-treated cancer and non-cancer patients. Based on the main logistic model, we obtained an estimate of OR equal to 2.55 [1.15; 5.65] for the pre-treated cancer patients. For the cancer patients without pre-treatment within 4 weeks, the OR is estimated at 1.68 [0.48; 5.87]. However, the overall effect of this variable is not associated with mortality.

**Table 3:** Management of patients during ICU stay.

	No cancer (n=315)	Cancer patients (n=105)	Hematological maligancies (n=51)	Solid tumors (n=54)
Time from ICU admission to oro-tracheal intubation, days	0 (0 - 0)	0 (0 - 1)	0 (0 - 3)	0 (0 - 1)
Oro-tracheal intubation, hours	360 (206 - 610)	360 (205 - 671)	504 (219 - 864)	306 (184 - 420)
Oxygen delivery method at ICU admission				
Nasal cannulae	27 (8·6%)	9 (8·6%)	4 (7·8%)	5 (9·3%)
Non-rebreather mask	51 (16·2%)	20 (19·0%)	13 (25·5%)	7 (13·0%)
Non-invasive ventilation	14 (4·5%)	2 (1·9%)	0 (0%)	2 (3·7%)
High-flow nasa-oxygen	120 (38·2%)	39 (37·1%)	17 (33·3%)	22 (40·7%)
Invasive machanical ventilation	102 (32·5%)	35 (33·3%)	17 (33·3%)	18 (33·3%)
Lower PaO2/FiO2 ratio during ICU stay	89 (65 - 130)	72 (57 - 105)	68 (57 - 100)	83 (57 - 110)
Prone positionning	152 (48·4%)	66 (62·9%)	36 (70·6%)	30 (55·6%)
Norepinephrine	157 (50·2%)	73 (69·5%)	38 (74·5%)	35 (64·8%)
Extra-corporeal membrane oxygenation	7 (2·2%)	4 (3·8%)	2 (3·9%)	2 (3·7%)
Antimicrobial treatment	256 (81·3%)	94 (89·5%)	49 (96·1%)	45 (83·3%)
Specific anti-SARS-CoV-2 treatment	230 (73·0%)	77 (73·3%)	39 (76·5%)	38 (70·4%)
Corticosteroids	189 (60·0%)	61 (58·1%)	29 (56·9%)	32 (59·3%)
Remdesivir	3 (1·0%)	2 (1·9%)	1 (2·0%)	1 (1·9%)
Ritonavir/Lopinavir	9 (2·9%)	2 (1·9%)	1 (2·0%)	1 (1·9%)
Hydroxychloroquin	12 (3·8%)	7 (6·7%)	5 (9·8%)	2 (3·7%)
Hydroxychloroquin + azithromycin	19 (6·0%)	5 (4·8%)	4 (7·8%)	1 (1·9%)
Plasma therapy	2 (0·6%)	2 (1·9%)	2 (3·9%)	0 (0%)
Polyvalent immunoglobulin	6 (1·9%)	2 (1·9%)	0 (0%)	2 (3·7%)

**Table 4:** Outcome of patients during their ICU stay.

	No cancer (n = 315)	Cancer patients (n = 105)	Hematological maligancies (n = 51)	Solid tumors (n = 54)
ARDS, Berlin definition	224 (71·1%)	81 (77·1%)	42 (82·4%)	39 (72·2%)
Superficial thrombosis	6 (1·9%)	2 (1·9%)	0 (0%)	2 (3·7%)
Deep thrombosis	24 (7·6%)	5 (4·8%)	3 (5·9%)	2 (3·7%)
Pulmonary embolism	21 (6·7%)	4 (3·8%)	4 (7·8%)	0 (0%)
ICU-acquired pneumonia	117 (37·1%)	47 (44·8%)	26 (51·0%)	21 (38·9%)
Arterial ischemia	1 (0·3%)	2 (1·9%)	0 (0%)	2 (3·7%)
Ancocoagulation therapy-related bleeding	20 (6·3%)	10 (9·5%)	6 (11·8%)	4 (7·4%)
ICU death at day 60	86 (27·4%)	59 (56·2%)	30 (58·8%)	29 (53·7%)
Main cause of ICU death				
Refractory hypoxemia	20 (23·3%)	13 (22·0%)	8 (26·7%)	5 (17·2%)
Withholding and withdrawing of lifesustaining therapies	24 (27·9%)	24 (40·7%)	14 (46·7%)	10 (34·5%)
Multiple organ failure	32 (37·2%)	17 (28·8%)	6 (20·0%)	11 (37·9%)
Pulmonary embolism	0 (0%)	1 (1·7%)	1 (3·3%)	0 (0%)
Arterial ischemia	0 (0%)	2 (3·4%)	0 (0%)	2 (6·9%)
ICU-acquired pneumonia	0 (0%)	1 (1·7%)	1 (3·3%)	0 (0%)
Others	6 (7·0%)	1 (1·7%)	0 (0%)	1 (3·4%)

ARDS, Acute respiratory distress syndrome; ICU, intensive care unit.

Finally, we computed a multivariate model including parameters significantly associated with mortality (cancer type, norepinephrine use, antimicrobial treatment use, and treatment with hydroxychloroquine and azithromycin). We added the age at admission in this model because this parameter has been widely and robustly reported in the literature as an independent parameter associated with mortality in patients with severe SARS-CoV-2 infection. Moreover, we added the period of admittance of the patient in this model based on the definition of the first and second waves of SARS-CoV-2 in France, with 1 August 2020 as the cut-off date (**Table 5**). The multivariate model identified that the presence of solid tumours (OR: 2·344, 95% CI: 0·87–6·31, p=0·062) and haematological malignancies (OR: 4·144, 95% CI: 1·24–13·83, p=0·062) were independently associated with mortality. Antimicrobial use and admission during the second wave were also independently associated with increased mortality in this study.

**Table 5:** Multivariate analysis of parameters associated with mortality.

Variable	OR [95%CI]	p-value
Solid tumors	2.344 (0.87-6.31)	0.0262
Hematological malignancies	4.144 (1.24-13.83)	0.0262
Age at admission > 70 y.o.	1.952 (0.94-4.04)	0.0717
Hydroxychloroquin + azithromycin combination treatment	0.316 (0.05-1.94)	0.2121
Antimicrobial treatment	6.345 (1.88-21.42)	0.0031
Admission after August, 1st 2021	2.608 (1.18-5.78)	0.0186
Norepinephrine use	3.561 (1.34-9.44)	0.0108

## Discussion

Among patients admitted to the ICU with severe SARS-COV-2 infection, patients with cancer represented 4% of the entire population in our study. Our analysis suggests that the risk of ICU death was 2·3-fold greater in cancer patients than in those without cancer after adjustment for confounding factors. Among cancer patients, those with haematological malignancies had a higher risk in comparison with solid tumours.

From the outset of the COVID-19 pandemic, cancer patients have been reported to be highly vulnerable (7, 8).. However, they account for a relatively low proportion, ranging from 2·4% to 9% of SARS-CoV-2 infection-related ICU admissions in series reporting this comorbidity (9–11).. This proportion is lower than the rate of patients admitted to a general ICU with cancer outside the COVID-19 context, which varies between 10·9% and 21·5% (12–14).. This difference does not seem to be related to a lower incidence of SARS-CoV-2 infection in patients with cancer, given that age-adjusted rates of SARS-CoV-2 infection in the population with cancer are reported to be similar to those in the population without cancer (15). Moreover, several studies have reported that patients with cancer are more likely to need ICU admission (16). Therefore, our result (i.e. the lower rate of cancer should be considered as an unfavorable comorbidity in the triage process. Moreover, in addition to prognosis, level of physiological compromise, and the existence of advanced directives among cancer patients; experts have also stated that ICU transfer decisions must include an assessment of the strain on ICU resources (17). However, a recent French study reported that differences in mortality rates at a regional level were not significantly associated with cancer status (18).

Owing to cancer-related immune alterations, immunosuppressive treatment, and cancer-related organ failure, patients with cancer have a higher risk of mortality in ICUs (19). Before the pandemic era, a meta-analysis of individual data of patients reported an overall ICU mortality rate of 49% in cancer patients, whereas the ICU mortality rate of patients without cancer ranges from 10% to 29% (20). In the context of COVID-19, a recent meta-analysis reported an overall mortality of 60% in 1,276 cancer patients admitted to the ICU with severe SARS-COV-2 infection, with an almost 2-fold increased risk of mortality than in patients without cancer (6).

Several parameters could play a role in the highly fatal ICU course of cancer patients with severe SARS-COV-2 infection. First, recent cancer treatment or comorbidities could arguably affect the course of SARS-CoV-2 infection. In our study, neither anticancer therapy in the 4 weeks before ICU admission nor comorbidities were significantly associated with the poor outcome of cancer patients, suggesting that the cancer itself exerts a deleterious effect on patients’ outcomes. Second, one could argue that cancer-related immune suppression could promote the occurrence of secondary infections and therefore contribute to a higher mortality rate. In line with this hypothesis, we identified a higher number of ICU-acquired infections in cancer patients in our study, particularly among patients with haematological malignancies. However, these nosocomial complications were considered responsible for only a low proportion of deaths, as assessed by physicians. These results must be interpreted cautiously, given controversial data regarding both the relatively low attributable mortality of nosocomial infections in septic patients and the variability in the definition of causes of death (21). Third, an increased risk of thromboembolic events could explain higher mortality rates among cancer patients. Cancer patients with SARS-CoV-2 infection accumulate cancer-related inflammatory pro-thrombotic risk along with the risk of thromboembolic events known to accompany viral infections (22). However, we noted only a few cases of pulmonary embolism and deep vein thrombosis. Moreover, cancer is not associated with a higher risk of a COVID-19-associated hypercoagulable state (23, 24). Finally, the impact of specific types of malignancy could influence the risk of death in patients with cancer. Although the sample size of this study does not allow the provision of robust statistical data regarding solid cancer subgroups, we found no differences in the repartition of solid cancer type. However, we observed a statistically higher risk of death in patients with haematological malignancies.

One of the more consistent findings in the literature is that there is a higher risk among patients with haematologic malignancies (25–27). In our study, patients with lymphoma and myeloma accounted for more than 80% of the population, with a low proportion of acute myeloid leukaemia. These patients exhibit a less pronounced immune response to the SARS-CoV-2 virus, including heterogeneous humoral responses and a high prevalence of prolonged virus shedding (28). However, in the specific context of lymphoma and chronic lymphoid leukaemia, it is still unclear whether altered B-cell immune response or B-cell depletion is the only cause of this increased mortality. Our results suggest that in the process of assessing whether a patient should be admitted to the ICU with severe SARS-CoV-2 infection, the existence of a haematological malignancy should be conceptualized differently from the presence of a solid tumor, as their prognosis varies significantly. Dan s

Using multivariate analysis, we also identified antimicrobial use and admission during the second wave as being independently associated with increased mortality. The association between antibiotics and mortality may reflect an increased incidence of secondary ICU-acquired pneumonia that was previously reported to exert a deleterious effect on the ICU course of COVID-19 (11). On the other hand, the independent deleterious effect of admission after 1 August 2020 was difficult to interpret. Though the case fatality rate of SARS-COV-2 infection between the first and second waves varies among countries and regions (29), studies suggest that changes in the proportion of SARS-COV-2 variant affect COVID-19 mortality (30).

This study had several limitations. First, the discrepancy we observed may involve characteristics of the cancer population that were not collected in this study, such as socioeconomic status or control of the underlying comorbidities. An additional limitation of this study relates to mortality attributable to SARS-CoV-2 infection. Mortality in cancer patients was assumed to be related to SARS-CoV-2 infection, whereas certain malignancies, such as acute myeloid leukaemia, are known to be life-threatening. Another limitation concerns the fact that despite the inverse probability of cancer weighting, some residual confounding factors could remain. Finally, the relatively small size of our cohort does not allow a statistically robust assessment of the effect of cancer subtypes on mortality.

## Conclusion

We identified a 2·3-fold increase in death rate between COVID-19 patients with and without cancer admitted to the ICU. Patients with haematological malignancies had a higher risk of death. Recent cancer-specific therapies, comorbidities, ICU management, ICU-acquired infections, and thromboembolic events were not associated with a higher risk of death, suggesting that the cancer itself exerts a deleterious effect on the clinical course of SARS-CoV-2 infection. Our results suggest reinforcing vaccination and prevention strategies in this specific population. Furthermore, strategies for ICU admission triage must consider this susceptibility and involve oncologists to deliver patient-centred care.

## Data Availability Statement

Clinical study report are available upon request, after approval by the study principal investigator (corresponding author). Deidentified individual participant data from this clinical trial, as well as data dictionary, can be requested by filling out the data request form for Gustave Roussy clinical trials at https://redcap.gustaveroussy.fr/redcap/surveys/?s=DYDTLPE4AM. The process is similar for every study sponsored by Gustave Roussy. The study steering committee and the sponsor will review the requests on a case-by-case basis. In case of approval, a specific agreement between the sponsor and the researcher may be required for data transfer.

## Ethics Statement

The studies involving human participants were reviewed and approved by Comité de protection des personnes Ile-de-Frande IV, IRB number 00003835. Written informed consent for participation was not required for this study in accordance with the national legislation and the institutional requirements.

## Author Contributions

J-FL, OM, and AB were responsible for trial conception and design. MLe, TC, PN, GP, RT, MJo, HP, LL, CD, LA, MJa, CG, KM, ML, BS, SS, AS, and J-FL recruited and cared for patients. Collection and assembly of data was done by J-FL, HP and AB, while the data analysis and interpretation was done by AB, NI, and J-FL. AB, NI, and J-FL had access to and verified the raw data. J-FL wrote the first draft of the manuscript and all authors participated in its writing and gave approval for publication. All authors had full access to all the data in the study and had final responsibility for the decision to submit for publication. All authors read and approved the final manuscript.

## Conflict of Interest

AB declare consulting fees for ROCHE SAS. J-FL is employed by bioMérieux but was employed by Institut Gustave Roussy when that study was performed.

The remaining authors declare that the research was conducted in the absence of any commercial or financial relationships that could be construed as a potential conflict of interest.

## Publisher’s Note

All claims expressed in this article are solely those of the authors and do not necessarily represent those of their affiliated organizations, or those of the publisher, the editors and the reviewers. Any product that may be evaluated in this article, or claim that may be made by its manufacturer, is not guaranteed or endorsed by the publisher.

## Abbreviations

ICU, intensive care unit, COVID-19, coronavirus disease 2019, SARS-CoV-2, severe acute respiratory syndrome coronavirus 2, BMI, body mass index, SAPSII, Simplified Acute Physiology Score 2, SOFA, Sequential Organ Failure Assessment, ARDS, Acute respiratory distress syndrome.
