# Supplementary material for: Outcomes of Patients With Active Cancer and COVID-19 in the Intensive-Care Unit: A Multicenter Ambispective Study
Source: Front Oncol. 2022 Mar 10;12:858276. doi: 10.3389/fonc.2022.858276 (PMC8960921; doi:10.3389/fonc.2022.858276)
Supplement: Supplementary file 1 [file DataSheet_1.doc]

**Study flow diagram**

**Analysis**

**1:3 ratio**

Patients admitted with severe SARS-CoV-2 infection in the 17 ICUs (n=2608)

Final cohort

(n=420)

Patients with cancer (solid tumor and/or haematological malignancies

(n= 105)

Patients with cancer (solid tumor and/or haematological malignancies

(n= 105)

Patients without cancer

(n=315)

Patients without cancer

(n=2503)
